# Characteristics of *Escherichia coli* Isolated from Intestinal Microbiota Children of 0–5 Years Old in the Commune of Abomey-Calavi

**DOI:** 10.1155/2022/6253894

**Published:** 2022-06-06

**Authors:** Haziz Sina, Durand Dah-Nouvlessounon, Tomabu Adjobimey, Bawa Boya, Ghislaine M. C. Dohoue, Christine N'tcha, Violette Chidikofan, Farid Baba-Moussa, Idrissou Abdoulaye, Adolphe Adjanohoun, Lamine Baba-Moussa

**Affiliations:** ^1^Laboratory of Biology and Molecular Typing in Microbiology, Department of Biochemistry and Cellular Biology, Faculty of Sciences and Techniques, University of Abomey-Calavi, 05 BP 1604 Cotonou, Godomey, Benin; ^2^Biochemistry and Molecular Biology Unit, Department of Biochemistry and Cellular Biology, Faculty of Sciences and Techniques, University of Abomey-Calavi, Godomey, Benin; ^3^Pediatrics Clinic of Abomey Calavi, Abomey-Calavi, Benin; ^4^Laboratory of Microbiology and Food Technology, Department of Plant Biology, Cotonou Benin, University of Abomey-Calavi, Godomey, Benin; ^5^University of Abomey-Calavi, Godomey, Benin; ^6^National Institute of Agronomic Research of Benin, Cotonou, Benin

## Abstract

*Escherichia coli* is a commensal bacterium and one of the first bacteria to colonize the digestive tract of newborns after birth. It is characterized by great versatility and metabolic flexibility that allows its survival in different niches. The present study aims at analyzing the diversity of *E. coli* strains isolated from the intestinal microbiota of children aged from 0 to 5 years in the commune of Abomey-Calavi in Benin. For this purpose, a descriptive and analytical cross-sectional study was conducted. A total of 135 stool samples were collected from the pediatric clinic of Abomey-Calavi. Microbiological analyses were performed according to standard microbiology analytical techniques. The molecular characterization of *E. coli* was performed by investigating eight genes (dinB, icdA, pabB, polB, putP, trpA, trpB, and uidA) using the PCR technique. The results showed that the average loading rate on stool samples was 3.74 × 10^7^ CFU/g for TAMF. A total of 7 species of bacteria were identified at different proportions: *Staphylococcus spp* (55.36%), *E. coli* (14.29%), *Klebsiella ornithinolytica* (12.5%), *Serratia odorifera* (5.36%), and *Enterobacter aerogenes* (5.36%). Interestingly, isolated *E. coli* presented a resistance of 100% to cefotaxime and aztreonam. In addition, resistances of 95.24% and 50% were observed against erythromycin and nalidixic acid, respectively. The molecular characterization of the isolated *E. coli* strains allowed us to discover another molecular variation within the isolated strains. Genes encoding the enzymes isocitrate dehydrogenase (icd) and DNA polymerase II (polB) were detected at 96.30% in the isolated *E. coli* strains. Moreover, the genes encoding the enzymes beta-D-glucuronidase (*uid*A) and DNA polymerase (dinB) were detected at 88.89% in the isolated *E. coli* strains. Interestingly, 81.48%, 85.19, 92.59%, and 100% of isolated *E. coli* strains expressed the genes encoding the enzymes tryptophan synthase subunit A (trpA), proline permease (*put*P), p-aminobenzoate synthase, and tryptophan synthase subunit B (trpB), respectively. The diversity of *E. coli* strains reflects the importance of regulatory mechanisms in the adaptation of bacteria to the gut microbiota.

## 1. Introduction

The gut microbiota is composed of more than 10^13^ bacteria, approximately the same number of cells that make up the human body [1]. It corresponds to the set of microorganisms in the digestive tract and performs three main and essential functions: metabolic, trophic, and defense [2]. Throughout the digestive tract, the diversity and abundance of the microbiota vary. Maximum bacterial abundance is reached in the distal colon with 10^11^ bacteria per gram of stool [3]. In recent years, the analysis of the composition and function of the gut microbiota has greatly expanded following the advent of new bacterial genome sequencing techniques. However, everyone has a unique gut microbiota in terms of bacterial species and relative proportions [4]. In adults, 90% of the gut microbiota is represented by the phyla Firmicutes and Bacteroidetes [5]. Therefore, it is admitted that *Escherichia coli* species belonging to the phylum of the Proteobacteria are not the main bacterial species within the human gut microbiota. *E. coli* spices represent typically making up less than 1% of the human gut microbiome [6].

In general, *E. coli* species are involved as commensal bacteria in the human gastrointestinal tract. This includes protection against epithelial cell injury [7], regulation of host fat storage [8], and stimulation of intestinal angiogenesis [9]. However, through the acquisition and combination of virulence and antibiotic resistance factors, these normally harmless commensal strains can become pathogenic and cause various diseases ranging from gastroenteritis to extraintestinal infections. Among intestinal *E. coli*, six intestinal pathovars have been described based primarily on the clinical signs generated and the pathogenicity factors expressed: enteropathogenic *E. coli* (EPEC), enteroinvasive *E. coli* (EIEC), enteroaggregative *E. coli* (EAgg or EAEC), enterotoxigenic *E. coli* (ETEC), diffusely adherent *E. coli* (DAEC), and enterohemorrhagic *E. coli* (EHEC) [10]. Extraintestinal pathogenic *E. coli* (ExPEC) strains are common pathogens, causing infections of variable severity [11].

Knowledge of the phylogeny of strains contributes to the recognition of their virulence potential and clinical outcome. ExPEC virulence factors are often grouped into islands associated with pathogenicity [12]. Pathogenic ExPEC strains belong to phylogenetic group B2 and, to a lesser extent, to the D group. In contrast, commensal isolates are assigned to groups A and B1. PCR is often used to assess phylogenetic groups [13]. The accuracy with which this method assigns strains to their correct phylogenetic group based on molecular characterization is good [14].

Traditional characterization, based on cultures [15], accounts for roughly 30% of the microorganisms observed and enumerated under the microscope. The global diversity of commensal gut bacteria species would be immense. In this regard, the use of molecular tools has indicated that the majority of the dominant bacterial species observed in an individual's fecal microbiota (about 80%) is specific to that individual [16]. Nowadays, the phylogenetic reassessment of the human gut microbiota is essentially limited to the dominant fraction, and our knowledge of subdominant bacteria, i.e., present at less than 10^8^ per gram of stool, may be incomplete and remains limited to cultivable isolates [15]. The ability to isolate and grow microorganisms *in vitro* remains a key step in acquiring knowledge, especially since phylogeny does not provide information on the *in situ* activity of microbes. Thus, the main objective of the present study is to investigate the genetic diversity of *E. coli* strains isolated from the intestinal microbiota of children aged 0 to 5 years in the commune of Abomey-Calavi.

## 2. Materials and Methods

### 2.1. Sampling and Sample Collection

The samples used in this study were randomly collected from 135 children of 0 to 5 years old in the Abomey-Calavi pediatric clinic, Benin. The Abomey-Calavi pediatric clinic is one of the biggest pediatric centers located in the biggest city of Benin according to the Population Stat, 2017–2022. The stool samples were collected between October and November 2020, period of children back to school. All the 135 stool samples were collected in a sterile identified bottle, without prior preparation, and transported to the laboratory, in ice (about 4°C). Once in the laboratory, all the collected samples were used for microbial analysis (Sections 2.2.1 and 2.2.2). During sampling, information was collected using a survey form. Collected data include the mode of delivery, the feeding mode (breast/bottle-feeding), antibiotic use, term of birth, and digestive disorders.

### 2.2. Microbiological Analyses

#### 2.2.1. Enumeration of Total Aerobic Mesophilic Flora

Each collected sample (10 g) was aseptically homogenized into sterile peptone water (90 ml). From this solution, a serial decimal dilution was made. For the detection and enumeration of total aerobic mesophilic flora (TAMF), dilutions 10^−1^ to 10^−3^ were sowed on Plate Count Agar (PCA) and incubated at 30°C for 72 h.

#### 2.2.2. Identification of Enterobacteriaceae

Eosin methyl blue agar (CM0069, OXOID, England) was used to isolate *Enterobacteriaceae*. The identification of such bacteria was made by the indole test and the Api20 E (bioMérieux, France) gallery [16].

#### 2.2.3. Antibiotic Susceptibility of Isolated *E. coli*

The susceptibility of isolates to 8 conventional antibiotic molecules was performed using the diffusion method [17]. The 8 antimicrobial agents (Oxoid, England) tested were as follows: erythromycin (E), cefotaxime (CTX), nalidixic acid (NA), aztreonam (ATM), imipenem (IPM), ciprofloxacin (CIP), ofloxacin (OFX), and norfloxacin (NOR).

### 2.3. Molecular Identification of *E. coli* Strains

#### 2.3.1. DNA Extraction

DNA extraction of all the isolated *E. coli* strains was performed following an adapted method of Rasmussen and Morrissey [18]. In brief, 1.5 ml tubes containing bacterial strains were centrifuged for 5 min at 12000 rpm. After discarding the supernatant, 500 *μ*l of sterile distilled water was added to the bacterial pellet. The mixture was then heated for 15 min at 95°C and centrifuged for 5 min at 12000 rpm. The obtained supernatants were recovered in new tubes, and 500 *μ*l of absolute ethanol was added before centrifugation for 5 min at 12000 rpm. The DNA pellets were suspended in 50 *μ*l sterile distilled water and maintained at 4°C.

#### 2.3.2. Molecular Characterization of *E. coli*

The presence of eight structural and/or housekeeping genes coding for essential proteins was used for this typing of isolated *Escherichia coli*. Thus, the eight genes (*dinB, icdA, pabB, polB, putP, trpA, trpB,* and *uidA*) were explored using the primer sequences provided in Table 1 [19]. For each targeted gene, the amplification reaction was performed on 25 *μ*l containing 12.5 *μ*l 2x GoTaq mix (PROMEGA, USA); 1 *μ*l primer F (10 *μ*M); 1 *μ*l primer R (10 *μ*M); and 3 *μ*l of DNA. The PCR amplification program was composed of an initial denaturation (94°C for 5 min); 35 cycles of denaturation cycles (94°C, 45°s); hybridization (52°C, 30°s) and elongation (72°C, 30°s); and a final elongation (72°C, 10 min).

### 2.4. Data Processing and Analysis

Data encoding was done using Microsoft Excel 2013 spreadsheet. Graph Pad Prism 8 (GraphPad software, Inc. USA) was used to make the graphs. In order to characterize *E. coli* species and genes, the presence or absence of 27 *E. coli* species in 8 genes was screened via DCA (detrended correspondence analysis) with the vegan package [20]. Finally, hierarchical ascending classification (HAC) was used to construct groups using R software (R Foundation for Statistical Computing, Vienna, Austria) version 3.6.1.

## 3. Results

### 3.1. Total Aerobic Mesophilic Flora Load of Stools

Table 2 shows the bacterial load according to the different stool samples analyzed. The total aerobic mesophilic flora average load rate was 3.74 × 10^7^ CFU/g. The stools of children in the 0–1-year age group were more loaded than those in the 4–5-year age group.

### 3.2. Microbial Diversity of Stools

**Figure 1:**
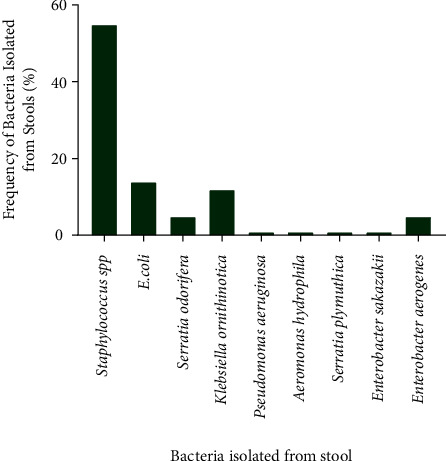
Overall distribution of strains isolated from stool.

Diversity of microbial species was observed in the stool samples analyzed. Indeed, 55.36% of the samples were contaminated by *Staphylococcus spp*. Gram-negative species were identified in different proportions, namely, *E. coli* (14.29%), *Serratia odorifera* (5.36%), *Klebsiella ornithinolytica* (12.5%), *Pseudomonas aeruginosa* (1.79%), *Aeromonas hydrophila* (1.79%), *Serratia plymuthica* (1.79%), *Enterobacter sakazakii* (1.79%), and *Enterobacter aerogenes* (5.36%).  Figure 1 shows the frequency of bacteria isolated from the 135 stool samples.

### 3.3. Susceptibility of Isolated Gram-Negative Bacteria to Antibiotics

**Figure 2:**
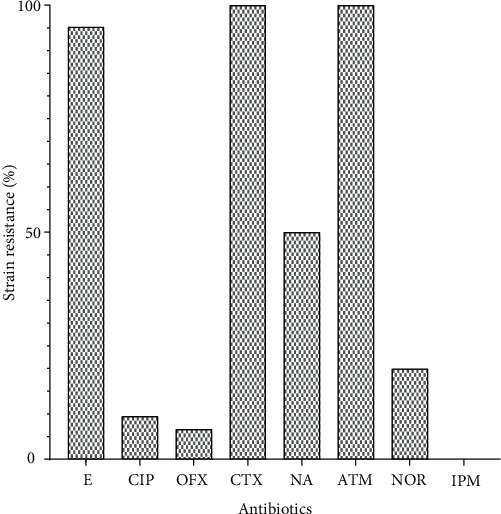
Resistance profile of isolated *E. coli* strains to antibiotics.

Susceptibility of *E. coli* isolates to antibiotics shows total resistance to cefotaxime and aztreonam, followed by resistance to erythromycin (95.24%) and nalidixic acid (50%) (Figure 2). The lowest resistance was observed with norfloxacin (20%), ciprofloxacin (9.52%), and ofloxacin (6.67%). All the isolates were sensitive to imipenem.

### 3.4. Molecular Variability of *E. coli* Strains

**Figure 3:**
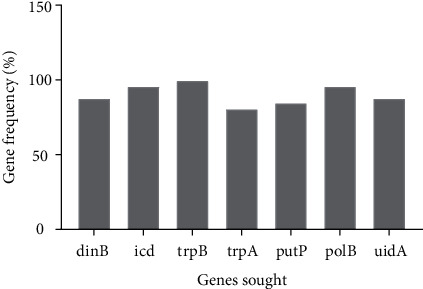
Percentage of *E. coli* household genes detected.

The search for household genes with the *E. coli* strains isolated from the stool samples allowed us to discover another molecular variation within the isolated strains. Thus, genes coding for the production of isocitrate dehydrogenase (icd) and DNA polymerase II (polB) were detected at 96.30%. In addition, the genes coding for the synthesis of beta-D-glucuronidase (uidA) and DNA polymerase IV (dinB) were detected at 88.89%. The isolated *E. coli* strains from stool harbor genes encode tryptophan synthase subunit A (trpA), proline permease (putP), p-aminobenzoate synthase, and tryptophan synthase subunit B (trpB) at 81.48%, 85.19, 92.59%, and 100%, respectively (Figure 3).

**Figure 4:**
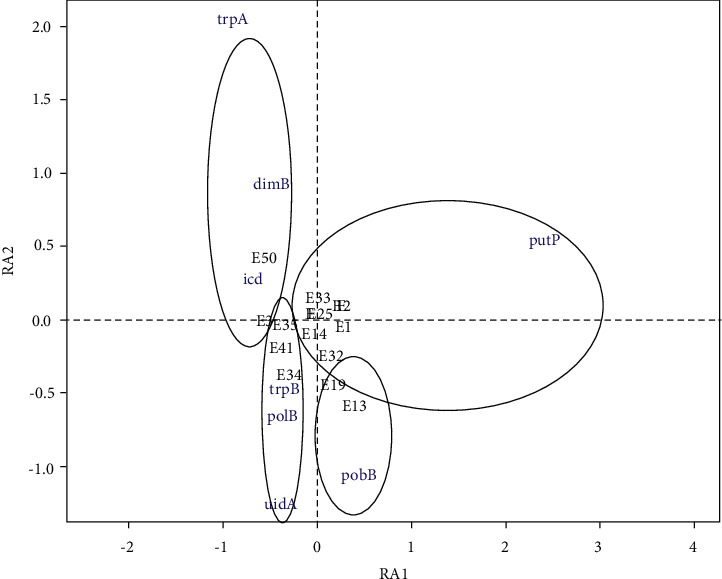
Projection on axes 1 and 2 of the DCA performed on the presence/absence data of 27 *E. coli* in 8 genes.

The DCA analysis performed on the matrix of presence/absence data of 27 *E. coli* species in 8 genes resulted in 4 groups (Figure 4). Group 1 (G1) is composed of 2 *E. coli* species, namely, E3 and E50, characterized by the presence of dinB and icd genes. Group 2 (G2) includes *E. coli* E13 and E19 and is distinguished from the others by the pabB gene. Group 3 (G3) is composed of *E. coli* strains isolated from samples E34, E35, E37, and E41 and is distinguished by the uidA, polB, and trpB genes. Group 4 (G4) is characterized by the remaining *E. coli* strains with the presence of the putP gene.

## 4. Discussion

The general objective of this study was to determine the genetic diversity of *E. coli* strains isolated from the intestinal microbiota of children aged 0–5 years in the commune of Abomey-Calavi. This study showed that 14.28% of the children sampled had digestive disorders. Morali [21] had already mentioned that functional digestive disorders in children are frequent reasons for seeking care [21]. Despite this frequency, pathophysiological knowledge is modest. Some disorders accompany physiological digestive maturation, and others represent adaptive responses to an overly strict educational process [21].

The microbiota refers to all the bacteria that inhabit and cohabit with humans, regardless of their anatomical location, on the skin, in the ear canal, bronchi, vaginal cavity, etc. [22]. However, research on these bacteria has mainly focused on the intestinal microbiota because they are found in higher numbers in the digestive tract. In addition, their influence on the organism's physiology seems to be decisive in the digestive tract. There are 100 billion bacteria in 1 gram of stool, as many as the cells that make up our brain [22]. In our study, the average load rate over one hundred and thirty-five (135) samples was 3.74 × 10^7^ CFU/g for TAMF.

It is often difficult to assess the diversity of the gut microbiota. However, microorganisms represent about 40% of the weight of the feces. There is, therefore, a fecal flora, which is easily accessible and analyzable. It includes a heterogeneous set of live and dead bacteria and yeasts from the resident and passing flora. It is often considered as an approximate reflection of the intestinal microbiota. The analysis of the fecal flora also makes it possible to detect the presence of pathogens or an imbalance of the colonic populations at the origin of digestive disorders [23]. In our work, of all the stool samples analyzed, 55.36% were contaminated by *Staphylococcus spp*, which also represents the overall contamination of the samples and 44.64% by enterobacteria. *Staphylococcus spp* is a frequent commensal bacterium of the human intestinal microbiota. In human pathology, ingestion of toxins produced ex vivo by the bacteria in insufficiently refrigerated food may be responsible for a brief digestive episode (less than 24 hours). In very debilitated settings, multiorgan infections with *Staphylococcus spp*, including diarrhea and/or colitis, have been written about [24]. In contrast, evidence of acute intestinal infections in the immunocompetent has never been made [24]. When *Staphylococcus spp* are at an abnormal number, especially *Staphylococcus aureus*, it can cause acute watery diarrhea of incubation, usually associated with vomiting and without fever; these signs heal in a few hours.

*Escherichia coli* is the second most isolated bacterium in our stool samples. The presence of numerous *E. coli* colonies in the stool, without further clarification, is normal and does not justify treatment on its own. Only the use of specific microbiological techniques allows the identification of pathogenic strains of *E. coli*, grouped into five pathovars (enterotoxigenic, enteropathogenic, enteroinvasive, enteroadherent, and enterohemorrhagic). According to Wang et al. [25], there is a link between the abnormal presence of *Escherichia coli* in the intestinal bacterial flora and the development of ulcerative colitis. In clinical routine, only testing for enterohemorrhagic *E. coli O157H7* by selective medium plating is indicated for hemorrhagic diarrhea, given the potential complications of this infection [26]. However, *E. coli* provides some benefits to the host by preventing colonization by certain pathogens such as *Salmonella* via production, among others, of bacteriocins. This host-bacteria interaction is close to mutualism [27, 28].

The *E. coli* strains isolated from our samples displays very high resistance to erythromycin (95.24%), ciprofloxacin (9.52%), ofloxacin (6.67%), cefotaxime (100%), nalidixic acid (50%), aztreonam (100%), and norfloxacin (20%). Although our study did not report many recent hospitalizations or antibiotic use among the child volunteers, we found a significant proportion of resistant bacteria in their fecal flora. For these children, many are likely to have been exposed to multiple courses of antibiotics due to the unrestricted or over-the-counter availability of antibiotics in developing countries [29]. There is a multiresistance of these strains to antibiotics belonging to different families, namely, *β*-lactams, aminoglycosides, fluoroquinolones, sulphonamides-trimethoprim, phenicoles, tetracyclines, and quinolones. This multiresistance observed in our strains is due to the probable presence of resistance genes [30]. This could have been caused by the uncontrolled use of antimicrobial agents. Such a high incidence of multidrug resistance can apparently prevail the replacement of antibiotic-susceptible microorganisms in the environment by those that are resistant to antibiotics [31]. However, among the antibiotics used, imipenem had total activity on *E. coli* isolates. This trend was also observed in Spain by Guembe et al. [32]. However, Hashemi et al. [33] found higher resistance rates of around 19% for imipenem. The absence of resistance to imipenem suggests that these molecules are still the treatment of choice against these bacteria.

We found a rate of resistance to ciprofloxacin (family of Fluoroquinolones) of 9.52%, which is slightly lower than the 12% reported in Morocco [34], 14% in Tunisia [35], and 12% in France [36]. Furthermore, it should be noted that drugs such as ciprofloxacin represent the first-line antibiotics commonly used by patients without a medical prescription. Since available treatment options are limited, antibiotic control policies and the implementation of infection control measures remain of great importance. We also found 50% resistance to nalidixic acid (Fluoroquinolone family). Fluoroquinolones are potent antibiotics and are most commonly prescribed for community-acquired urinary tract infections. A relationship between increased prescriptions of quinolones and an increase in bacterial resistance has been reported in several countries [37, 38].

These results can be explained by the impact of these different classes of antibiotics on the fecal flora, as described by other authors [39, 40]. Antibiotic pressure is a major determinant of the emergence and dissemination of multidrug-resistant bacteria [41]. Unsuitable initial probabilistic antibiotic therapy in this cohort of frail patients would seem to be a risk factor for severe infection, which would suggest the use of broad-spectrum antibiotic treatment from the first sign of infection in these patients, even if a small number of patients are concerned [42] by unsuitable initial antibiotic therapy, does not allow any definitive conclusion. However, this same prescription of broad-spectrum antibiotics represents a risk factor for the occurrence of bacteremia in the 6 months following its administration. Therefore, the importance of the practitioner's clinical sense in the decision to start antibiotic therapy (broad spectrum from the outset, targeted antibiotic therapy, or armed standby) and the use in these specific situations of probabilistic antibiotic therapy that does not impact the flora (for example, aminoglycosides) must be studied prospectively.

Molecular characterization was performed to identify *E. coli* strains by targeting eight genes. The trp B and trp A genes (or trp BA genes) coexist in the tryptophan operon of the *E. coli* genome [25]. In our study, all *E. coli* strains isolated from stool contain the genes encoding the enzyme tryptophan synthase subunit B (trpB) and 81.48% tryptophan synthase subunit A (trpA). Strains that lack the tryptophan synthase subunit A (trpA) gene may be due to poor gene expression in *E. coli.* In addition, it may be due to inefficient translation initiation. Inefficient translation initiation in *E. coli* occurs in many adenine- and thymine-rich genes [43].

The *β*-D-glucuronidase, encoded by the uidA gene, is commonly used to specifically identify *E. coli* [44]. The beta-glucuronidase enzyme was detected in 88.89% of *Escherichia coli* strains isolated from our stool samples. Our results are lower than those obtained by Martins et al. [45], where 97.7% of the *Escherichia coli* strains possessed the uidA gene. This difference could be explained by the mutation of the uidA structural gene because two mutations in the uidA structural gene explain the lack of glucuronidase activity in *Escherichia coli* strains [44].

In *Escherichia coli*, flux through the shunt is controlled by regulating isocitrate dehydrogenase [46]. In our study, genes encoding the enzyme isocitrate dehydrogenase (icd) were detected at 96.30%. Isocitrate dehydrogenase (icd) is an enzyme involved in the regulation of tumorigenesis [47]. Isocitrate dehydrogenases (icd) are enzymes that oxidize isocitrate to *α*-ketoglutarate. There are several isoenzymes depending on the cellular compartments, including a mitochondrial form with NAD+ that participates in the Krebs cycle and a cytoplasmic form with NADP+ that participates in lipogenesis. The fate of the isocitrate molecule in *Escherichia coli*, either through the glyoxylate shunt or the TCA cycle, is regulated by phosphorylation of isocitrate dehydrogenase by phosphatase kinase AceK [46].

## 5. Conclusion

This study provides important data on the molecular characteristics of *E. coli* strains isolated from the children stool. Significant diversity of microbial species was observed in the 135 analyzed stool samples. The identified microbial species were as follows: *Staphylococcus spp., E. coli, Serratia odorifera, Klebsiella ornithinolytica, Pseudomonas aeruginosa, Aeromonas hydrophila, Serratia plymuthica, Enterobacter sakazakii,* and *Enterobacter aerogenes.* The highlighting of the susceptibility to antibiotics conventionally used in hospitals revealed the multiresistance to antibiotics belonging to different families. The molecular characterization of *E. coli* strains isolated from the stool samples revealed another molecular variation within the isolated strains. It will thus be useful to extend the period and the study area in aim to draw more global and relevant conclusion.

## Figures and Tables

**Table 1:** Sequences of primers [19] used for characterization of isolated *Escherichia coli* strains.

Target Genes	Function	Sequence (5′⟶3′)
*dinB*	DNA polymerase	F: TGAGAGGTGAGCAATGCGTA
		R: CGTAGCCCCATCGCTTCCAG

*icdA*	Isocitrate dehydrogenase	F: ATTCGCTTCCCGGAACATTG
		R: ATGATCGCGTCACCAAAYTC

*pabB*	*p*-Aminobenzoate synthase	F: AATCCAATATGACCCGCGAG
		R: GGTTCCAGTTCGTCGATAAT

*polB*	Polymerase PolII	F: GGCGGCTATGTGATGGATTC
		R: GGTTGGCATCAGAAAACGGC

*putP*	Proline permease	F: CTGTTTAACCCGTGGATTGC
		R: GCATCGGCCTCGGCAAAGCG

*trpA*	Tryptophan synthase subunit A	F: GCTACGAATCTCTGTTTGCC
		R: GCTTTCATCGGTTGTACAAA

*trpB*	Tryptophan synthase subunit B	F: CACTATATGCTGGGCACCGC
		R: CCTCGTGCTTTCAAAATATC

*uidA*	*β*-Glucuronidase	F: CATTACGGCAAAGTGTGGGTCAAT
		R: CCATCAGCACGTTATCGAATCCTT

**Table 2:** Average microbial load of children's stools by age.

Age	Average microbial load
[0–2]	5.49 × 10^7^
[2–3]	3.16 × 10^7^
[3–4]	4.23 × 10^7^
[4–5]	2.82 × 10^7^

## Data Availability

The data used to support the findings of this work are available from the corresponding author upon request.

## Abbreviations

ATM: Aztreonam
CFU: Colony forming unit
CIP: Ciprofloxacin
CTX: Cefotaxime
dinB: Gene encoding for DNA polymerase
E: Erythromycin
G1: Group 1
G2: Group 2
G3: Group 3
G4: Group 4
icdA: Gene encoding for isocitrate dehydrogenase
IPM: Imipenem
NA: Nalidixic acid
NOR: Norfloxacin
OFX: Ofloxacin
pabB: Gene encoding for p-aminobenzoate synthase
PCA: Plate Count Agar
PCR: Polymerase chain reaction
polB: Gene encoding for polymerase PolII
putP: Gene encoding for proline permease
TAMF: Total aerobic mesophilic flora
TCA cycle: Tricarboxylic acid cycle
trpA: Gene encoding for tryptophan synthase subunit A
trpB: Gene encoding for tryptophan synthase subunit B
uidA: Gene encoding for *β*-glucuronidase.
